# Oneself is more important: Exploring the role of narcissism and fear of negative evaluation in the relationship between subjective social class and dishonesty

**DOI:** 10.1371/journal.pone.0218076

**Published:** 2019-06-07

**Authors:** Song Wu, Jingyuan Liang, Jing Lin, Wei Cai

**Affiliations:** 1 College of Psychology and Sociology, Shenzhen University, Shenzhen, China; 2 School of Humanities and Management / Research Center for Quality of Life and Applied Psychology, Guangdong Medical University, Dongguan, China; Middlesex University, UNITED KINGDOM

## Abstract

Previous studies have found that high social class individuals are more dishonest than low social class ones. However, the underlying mechanism of this phenomenon is still unclear. The “ignoring negative consequences” hypothesis suggests that belonging to a high social class makes individuals ignore the negative consequences of dishonesty, whereas the “self-focused” hypothesis suggests that belonging to a high social class makes individuals focus more on the self and self-interests. The present study aims to examine these two hypotheses by measuring participants’ subjective social class, narcissism, fear of negative evaluation, and tendency to be dishonest. To this end, an online survey was conducted. Results provide evidence for the self-focused hypothesis by showing that subjective social class positively predicts the tendency to be dishonest, and narcissism plays a mediating role in this relationship.

## Introduction

Most of the time, dishonesty—a common social behavior—can undermine social trust and lead to huge losses for both individuals and collectives. A survey has found that about 40% of people tell at least one lie in a 24-hour period [1], but people can detect lies at an accuracy level that is just above the random level [2, 3]. Therefore, knowing when and why people practice dishonesty is helpful for avoiding the negative consequences of dishonesty. Studies have found that both personal and environmental factors can influence dishonesty, such as gender [4], ego depletion [5], time pressure [6, 7], and air pollution [8]. The present study focuses on an important factor in social life: social class.

Social class has a powerful impact on individuals’ social attitudes and behaviors. Although individuals across all social classes may sometimes act unethically or dishonestly, previous studies have found that higher social class individuals tend to exhibit more unethical behaviors than do lower social class ones, especially in moderate and self-beneficial situations [9, 10]. However, the underlying mechanism of this phenomenon is still unknown. Therefore, the purpose of the present study is to provide some insight into this mechanism.

Generally speaking, social class refers to one’s relative standing based on wealth and education in a certain society [11]. Higher social class individuals usually have more wealth, a better education, and higher status occupations; therefore, they are typically regarded as being well-behaved and role models in a society. However, previous studies have found that high social class can significantly increase practice of unethical behaviors [9, 10]. In particular, researchers [10] found that higher social class individuals are more likely to violate traffic regulations (Studies 1 & 2), report higher tendencies to act unethically (Studies 3, 4, & 7), and lie to gain more monetary rewards (Study 6). These results are consistent with other studies [9, 12]. However, there is still no definite conclusion concerning why high social class promotes unethical behaviors. Based on previous results, we summarized two plausible underlying mechanisms.

First, since higher social class individuals usually have more resources, they are more independent and less likely to be constrained by structural factors [13]. Specifically, belonging to a high social class can repress individuals’ moral identity and cause them to underestimate the moral risk of unethical behaviors [14]. Furthermore, their plentiful resources can offset the potential cost of unethical behaviors, and they may care less about negative evaluation and pressure from others [15, 16]. In other words, higher social class individuals are less likely to be affected by the negative consequences of unethical behaviors; we called this “ignoring negative consequences” hypothesis.

Second, some researchers have also proposed that higher social class individuals may feel powerful, have an agentic orientation, and focus more on the self, whereas lower social class individuals may feel powerless, have a communal orientation, and focus more on others [17, 18]. More specifically, belonging to a higher social class may make individuals more selfish and focused on self-interests, pursuing such interests with fewer restrictions [19, 20]. By contrast, belonging to a lower social class makes individuals more interdependent, more focused on others, and less likely to harm others [17, 21]. In other words, higher social class individuals are more likely to pursue self-goals through any possible means, including unethical behaviors; we called this “self-focused” hypothesis.

The latter explanation—the self-focused hypothesis—has been supported by some studies. For example, higher social class individuals were found to be less generous, charitable, trusting, and helpful than lower social class ones [22]. In addition, a study found that higher social class individuals practiced more dishonesty that benefited themselves, whereas lower social class ones practiced more dishonesty that benefited others [9]. Taken together, these results suggest that belonging to a higher social class makes individuals more self-focused. The present study aims to provide more evidence for the self-focused hypothesis by examining the role of fear of negative evaluation (FNE) and narcissism in the relationship between social class and dishonesty.

FNE refers to “the degree to which people experience apprehension at the prospect of being evaluated negatively” [23]. FNE plays an important role in interpersonal interactions, especially in a potential evaluation situation. Individuals with high FNE are more sensitive to negative social cues [24], which have a powerful impact on approval-seeking behaviors, such as conforming and prosocial behaviors [25, 26]. Dishonest behaviors are usually not socially accepted and, if they are detected, may cause unpleasant consequences, such as negative evaluation from others. Therefore, individuals with high FNE may be less likely to exhibit dishonest behaviors. In this regard, an early study found that FNE is negatively associated with cheating [27]. In addition, previous studies have suggested that social class could negatively predict individuals’ scores on the FNE scale [15, 28]. Based on these findings, the ignoring negative consequences hypothesis is likely to be supported by confirming the mediating role of FNE in the relationship between social class and dishonesty.

Narcissism refers to the tendency to exaggerate self-importance and be dominant, exploitative, and feel entitled [29]. In the present study, we regarded narcissism as a normal personality trait (i.e., grandiose narcissism) rather than as a personality disorder (i.e., vulnerable narcissism). Previous studies have found that individuals with high narcissism think they are better than others, perceive themselves to be unique, and act more selfishly [30–33]. These findings suggest that self-focus is an essential component of narcissism. Since previous studies have found that social class can positively predict narcissism [34], it is reasonable to assert that high social class individuals tend to be more self-focused. In addition, there is a positive association between narcissism and dishonest behaviors. In this regard, studies have shown that individuals with high narcissism report more positive attitudes toward dishonesty [35] and exhibit more dishonest behaviors [36]. Therefore, the self-focused hypothesis is likely to be supported by confirming the mediating role of narcissism in the relationship between social class and dishonesty.

In summary, the present study aimed to examine the ignoring negative consequences hypothesis and the self-focused hypothesis by measuring participants’ subjective social class, narcissism, FNE, and tendency to be dishonest. We hypothesize that subjective social class will significantly and positively predict tendency to be dishonest (Hypothesis 1). However, we also propose two exploratory hypotheses: first, FNE will mediate the relationship between subjective social class and tendency to be dishonest (Hypothesis 2); second, narcissism will mediate the relationship between subjective social class and tendency to be dishonest (Hypothesis 3). Since the ignoring negative consequences hypothesis and the self-focused hypothesis are not mutually exclusive, both hypotheses 2 and 3 may be supported by the data.

## Materials and method

This study was a part of a large project which was approved by the ethics review board of Shenzhen university. Participants completed this study anonymously, and no identification information was collected. Before the formal study, participants were informed that this is a scientific study and that they could withdraw at any time. Since this study was completed online, participants’ consent was obtained by clicking the “confirm” label. Only two kinds of individuals were eligible to participate in the study: (1) those who were aged over 18, and (2) those who were aged between 16–18 but lived on their own. According to the Chinese law, the second kind of people are also classed as adults, and so we were not required to obtain consent from their parents.

### Participants

Participants completed all measurements online by clicking a link. The authors first posted this link as a “moment” on their personal internet social networking platform (i.e., WeChat), inviting adults to take part in our study voluntarily. At the same time, authors also asked friends to share this “moment” to make it available to more people. The sampling was stopped once the number of valid participants exceeded 400. A total of 400 people (253 females and 147 males) participated in our study, whose ages ranged from 17 to 62 years (*M* = 32.19, *SD* = 10.26).

### Measurements

#### Subjective social class

Subjective social class was measured by a traditional self-report one-item scale [37]. Participants were presented with a picture of a 10-step ladder and were told that the ladder represented where people stand in society, with the top of the ladder representing people belonging to the highest social class and the bottom representing people belonging to the lowest social class. Participants were asked to select where they would place themselves on this ladder (1 to 10). We believe this subjective measurement is a reliable indicator of social class in the present study because of two reasons. First, previous studies have used this measurement to examine the relationship between social class and moral behaviors and found that its predictive effect is similar to that of the objective measurements [9, 10, 22]. For example, Dubois and colleagues found that the subjective social class (Experiments 1 and 3) and actual income (Experiment 2) have similar predictive effects on unethical behaviors [9]. Moreover, Piff and colleagues also found that the subjective social class (Studies 3, 5, and 6) have a consistent predictive pattern with vehicle value (Studies 1 and 2) and manipulation of social class (Study 4) on unethical behaviors [10]. Second, previous studies found that sometimes subjective social class is a better indicator than objective social class, especially when there is a gap between subjective and objective social class [38, 39]. Since participants in the present study included college students, the subjective social class was a more appropriate measurement than the objective one.

#### Narcissism

Narcissism was measured using the Narcissistic Admiration and Rivalry Questionnaire, NARQ [40]. There is evidence showing that the NARQ is also suitable for the Chinese culture [41]. The NARQ consists of 18 items, such as “I deserve to be seen as a great personality.” Participants were asked to rate the extent to which they agreed with each item on a 7-point scale (1 = *totally disagree*, 7 = *totally agree*). The mean score of all 18 items was the indicator of narcissism, with higher scores suggesting higher narcissism. The Cronbach’s alpha for all 18 items was 0.90.

#### FNE

FNE was measured using the Brief Fear of Negative Evaluation-II, BFNE-II [42]. BFNE-II consists of 12 items, such as " I worry about what other people will think of me even when I know it doesn’t make any difference." In the present study, participants were asked to rate to the extent to which they agreed with each item on a 7-point scale (1 = *totally disagree*, 7 = *totally agree*). The mean score of all 12 items was the indicator of FNE, with higher scores suggesting higher FNE. The Cronbach’s alpha for all 12 items was 0.97.

#### Dishonesty

Dishonesty was measured by using the Self-Reported Inappropriate Negotiation Strategies (SINS) scale [8, 43]. We only selected 12 items from the original SINS scale because not all items describe an obvious dishonesty. Participants were asked to imagine that they were engaging in a negotiation that was very important to them and their business, and each item described a tactic that was available for use in this negotiation (e.g., Get the other party to think that I like him/her personally despite the fact that I don’t really). Participants were instructed to indicate the possibility to which they would use each tactic in such a situation on a 7-point scale (1 = *not at all*, 7 = *very*; Cronbach’s α = .94). The mean score of all 12 items was the indicator of dishonesty, with higher scores suggesting higher tendencies to be dishonest.

### Statistical analysis

Correlation coefficients were first calculated between all measurements. Then two hierarchical linear models were performed to separately analyze the mediating effect of narcissism and FNE in the relationship between subjective social class and dishonesty. For both models, a bootstrap analysis was also performed to examine the significance of both indirect effects.

## Results

The intercorrelations and descriptive statistics of subjective social class, narcissism, FNE, and dishonesty are shown in Table 1. We found a significant positive correlation between subjective social class and dishonesty and a significant positive correlation between subjective social class and narcissism. However, the correlation between subjective social class and FNE was not significant.

**Table 1:** Summary of intercorrelations and descriptive statistical results of all scales.

	1	2	3	4
1. SSC	-			
2. Narcissism	0.22**	-		
3. FNE	-0.02	-0.50**	-	
4. Dishonesty	0.12*	0.58**	-0.40**	-
*M*	4.60	3.37	3.87	3.28
*SD*	1,76	1.01	1.48	1.33
Cronbach’s alpha	N/A	0.90	0.97	0.94

*Notes*. For all scales, *N* = 400. SSC = subjective social class, FNE = fear of negative evaluation.

*p < .05

**p < .01

### Gender differences

An independent-samples t-test was performed to examine potential gender differences in all measurements. Results showed that males reported higher scores on narcissism and dishonesty than females, but there were no gender differences for subjective social class and FNE (Table 2).

**Table 2:** The analyses of gender differences for all scales.

	Male		Female		Independent-samples T test		
	*M*	*SD*	*M*	*SD*	*t*	*p*	*d*
SSC	4.76	1.92	4.50	1.65	1.39	.165	0.15
Narcissism	3.63	1.15	3.21	0.88	3.86	< .001	0.41
FNE	3.92	1.50	3.84	1.48	0.53	.594	0.05
Dishonesty	3.60	1.40	3.10	1.26	3.67	< .001	0.38

*Notes*. For all scales, *N* = 400. SSC = subjective social class, FNE = fear of negative evaluation.

### Mediation analysis of narcissism

**Fig 1:**
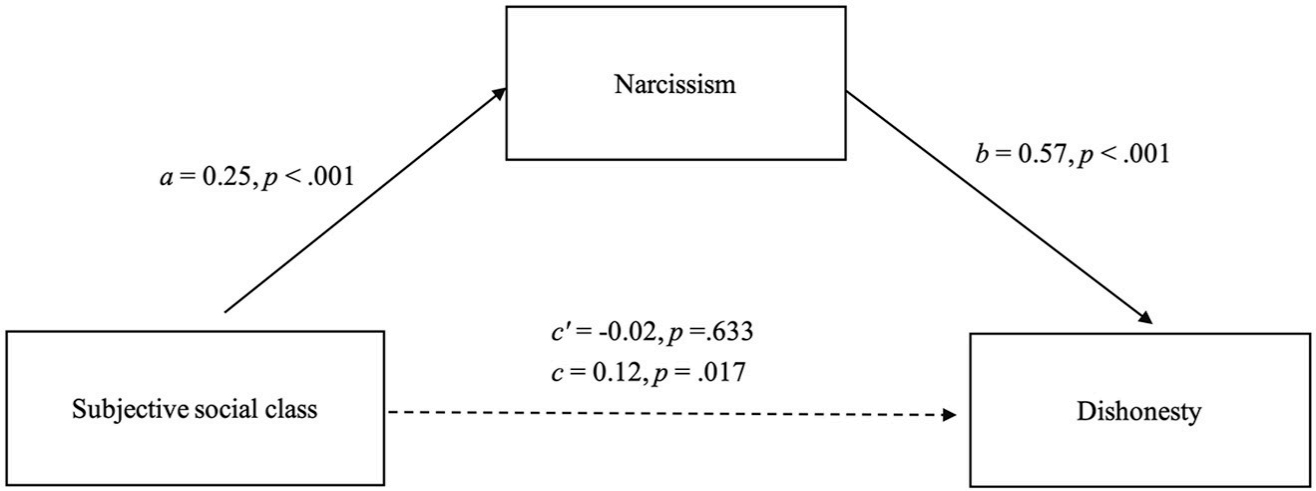
The mediating effect of narcissism on the relationship between subjective social class and dishonesty.

To examine the mediating effect of narcissism on the relationship between subjective social class and dishonesty, three regression analyses were conducted (see Fig 1). In all analyses, gender and age were included as control variables. In step one, subjective social class significantly and positively predicted dishonesty, *β* = 0.12, *t* = 2.40, *p* = .017; in step two, subjective social class significantly and positively predicted narcissism, *β* = 0.25, *t* = 5.08, *p* < .001; and in step three, after adding subjective social class and narcissism into the regression, results showed that narcissism still significantly and positively predicted dishonesty, *β* = 0.57, *t* = 13.17, *p* < .001, but the effect of subjective social class on dishonesty was not significant, *β* = -0.02, *t* = -0.48, *p* = .633. A bootstrap analysis (5,000 bootstrapping samples) found that the indirect effect was 0.11, *SE* = 0.03, 95% confidence intervals (CI) = [0.05, 0.16]. Because the 95% CI did not include zero, this suggests that the relationship between subjective social class and dishonesty was completely mediated by narcissism.

### Mediation analysis of FNE

Although the correlation between subjective social class and FNE was not significant, we also examined the mediating effect of FNE on the relationship between subjective social class and dishonesty. As in the previous analysis, subjective social class significantly and positively predicted dishonesty, but not FNE, *β* = -0.06, *t* = -1.27, *p* = .206. Finally, when both subjective social class and FNE were added into the regression, the predicting effect of FNE was significant, *β* = -0.41, *t* = -8.88, *p* < .001, and the predicting effect of subjective social class was still significant, *β* = 0.09, *t* = 2.05, *p* = .041. However, the bootstrap analysis (5,000 bootstrapping samples) found that the indirect effect was 0.02, SE = 0.02, 95% CI = [-0.01, 0.05]. Because the 95% CI included zero, this suggests that the relationship between subjective social class and dishonesty was not mediated by FNE.

### Moderating analysis of gender

In order to examine the moderating effect of gender on the relationship between subjective social class and dishonesty, we submitted subjective social class, gender, and product of social class and gender to a linear regression analysis with dishonesty as the dependent variable. Results showed that the moderating effect of gender was not significant, *β* = 0.06, *t* = 1.15, *p* = .251. A similar analysis was performed to examine the moderating effect of gender on the relationship between subjective social class and narcissism and was still not significant, *β* = -0.003, *t* = -0.06, *p* = .954.

## Discussion

Based on the results, Hypothesis 1 was supported in that participants’ subjective social class positively predicted their dishonest tendencies. This finding was consistent with previous studies that measured actual dishonest behaviors in real life or laboratory situations [9, 10]. We also examined the underlying mechanism of the relationship between subjective social class and dishonesty. FNE was measured to examine the ignoring negative consequences hypothesis, and narcissism was measured to examine the self-focused hypothesis. Although both FNE and narcissism were significantly associated with the tendency to be dishonest, we found a significant mediating effect only for narcissism. That is to say, Hypothesis 3 and the self-focused hypothesis were supported. Narcissism is regarded as an indicator of self-focus, and individuals with high narcissism are more likely to focus on themselves than on others [30, 33, 40]. It is reasonable to speculate that high subjective social class individuals are more self-focused and thus they put more effort into pursuing self-interests. Therefore, individuals with high subjective social class practice dishonesty when it supports their self-interests, but not otherwise. This could explain why high social class individuals practice less prosocial behaviors and prosocial lies [9, 22]. Since previous studies found that males tend to be more dishonest and narcissistic than females [4, 44], one may argue that gender may also account for the mediating role of narcissism in the relationship between subjective social class and dishonesty. However, this explanation can be excluded based on our analyses. Although we found significant gender differences in narcissism and dishonesty, the gender differences in subjective social class and the moderating effect of gender were not significant. Therefore, we believe that gender differences do not weaken the self-focused hypothesis.

By contrast, we did not find a significant correlation between subjective social class and FNE. This suggests that high subjective social class individuals do not ignore negative consequences, at least in the form of negative evaluation. Although the present study did not provide evidence for the ignoring negative consequences hypothesis (Hypothesis 2), the paucity of relevant research does not enable us to draw a definite conclusion.

### Limitations and future directions

The study’s main limitation was that it only obtained correlational results from which we cannot reach causal conclusions. One may argue that the relationship between subjective social class and dishonesty is accounted for by effects (e.g., Dunning–Kruger effect or illusory superiority effect) whereby individuals tend to overestimate their cognitive skills or other traits [45, 46]. However, we believe this is not the case. Specifically, if there was a Dunning-Kruger effect or illusory superiority effect in the present study, following a logical approach, participants would have inflated themselves by reporting a higher subjective social class and less dishonesty. Thus, we would have been more likely to obtain a negative relationship between subjective social class and dishonesty. On the contrary, the results showed a significant positive relationship between subjective social class and dishonesty.

Although previous studies have examined the causal link between social class and dishonesty [9, 10], no studies have examined the mediating effect of narcissism by manipulating social class. However, previous studies provided indirect evidence by showing that high social class individuals practice less dishonesty that benefits others than do low social class ones [9]. Future studies should use well-controlled experiments to examine the self-focused hypothesis, such as by manipulating social class and measuring actual dishonest behaviors.

Another limitation of our study is that we only considered negative evaluation as a negative consequence of dishonesty. However, negative evaluation from others is a relatively implicit and less serious consequence; dishonesty may cause other more explicit and severe consequences, such as social exclusion, monetary loss, and even legal penalties. It is possible that belonging to a high social class makes individuals ignore these explicit consequences rather than negative evaluation; that is, they are more concerned about the latter. Therefore, future studies could measure or manipulate these consequences to examine in greater depth the ignoring negative consequences hypothesis.

Finally, no actual dishonest behaviors were measured in the present study. Since a self-reported tendency to be dishonest may not always predict actual behavior, more studies on actual dishonesty are needed to examine the mediating role of narcissism in the relationship between subjective social class and dishonesty.
